# Porous Silicon Bragg Reflector/Carbon Dot Hybrids: Synthesis, Nanostructure, and Optical Properties

**DOI:** 10.3389/fchem.2018.00574

**Published:** 2018-11-23

**Authors:** Naama Massad-Ivanir, Susanta Kumar Bhunia, Raz Jelinek, Ester Segal

**Affiliations:** ^1^Department of Biotechnology and Food Engineering, Technion–Israel Institute of Technology, Haifa, Israel; ^2^Schulich Faculty of Chemistry, Technion–Israel Institute of Technology, Haifa, Israel; ^3^Department of Chemistry, Ben Gurion University of the Negev, Beer Sheva, Israel; ^4^Ilse Katz Institute for Nanotechnology, Ben Gurion University of the Negev, Beer Sheva, Israel; ^5^The Russell Berrie Nanotechnology Institute, Technion–Israel Institute of Technology, Haifa, Israel

**Keywords:** porous silicon, carbon dots, Bragg reflectors, photoluminescence, fluorescence, Fabry–Pérot, optical properties

## Abstract

Carbon dots (C-dots) exhibit unique fluorescence properties, mostly depending upon their physical environments. Here we investigate the optical properties and nanostructure of Carbon dots (C-dots) which are synthesized *in situ* within different porous Silicon (PSi) Bragg reflectors. The resulting hybrids were characterized by photoluminescence, X-ray photoelectron, and Fourier Transform Infrared spectroscopies, as well as by confocal and transmission electron microscopy. We show that by tailoring the location of the PSi Bragg reflector photonic bandgap and its oxidation level, the C-dots emission spectral features can be tuned. Notably, their fluorescence emission can be significantly enhanced when the high reflection band of the PSi host overlaps with the confined C-dots' peak wavelength, and the PSi matrix is thermally oxidized at mild conditions. These phenomena are observed for multiple compositions of PSi Bragg reflectors/C-dots hybrids.

## Introduction

In recent years, carbon dots (C-dots) have attracted considerable attention as a promising green nanomaterial for applications in sensing, bio-imaging, and optical devices owing to their unique optical properties (Lim et al., 2015; Tuerhong et al., 2017; Wang et al., 2017). Specifically, C-dots exhibit excitation-dependent emission, bright luminescence, low bleaching, and biocompatibility (Baker and Baker, 2010; Ding et al., 2014; Bhunia et al., 2016a; Sun and Lei, 2017). C-dots are small carbonaceous nanoparticles, with a typical size below 10 nm, and they are easily synthesized from various inexpensive and available precursors (Bhunia et al., 2016a,b). A notable feature of C-dots is the dependence of their optical properties upon their surface structure and proximate physical environments (Kwon et al., 2013; Zhang et al., 2013; Nandi et al., 2014).

Porous silicon (PSi)-based nanostructures have been widely reported as potential host matrices for light emitting materials, including organic dyes (Palestino et al., 2008; Jenie et al., 2014, 2015; Krismastuti et al., 2014; Mo et al., 2017) and quantum dots (QDs) (DeLouise Lisa and Ouyang, 2009; Qiao et al., 2010; Gaur et al., 2011, 2013; Dovzhenko et al., 2015, 2018a; Liu et al., 2015; Dovzhenko D. S. et al., 2016; Li et al., 2017; Zhang et al., 2017). PSi-based photonic crystals hosts [e.g., Bragg reflector (Liu et al., 2015; He et al., 2017; Li et al., 2017) and microcavities (Jenie et al., 2014, 2015)] have been shown to affect the propagation and distribution of the light emitted by the guest fluorophores (Pacholski, 2013; Dovzhenko D. et al., 2016; Dovzhenko D. S. et al., 2016). Specifically, PSi-based microcavities have been shown to improve the spectral properties of emitting molecules, e.g., quantum yield, photostability and luminescence lifetime, by alignment between the reflectance spectrum dip of the microcavity and the emission of the fluorophores (Jenie et al., 2016).

We have recently synthesized a new hybrid host–guest material, consisting of a Fabry–Pérot PSi thin film encapsulating C-dots (Massad-Ivanir et al., 2018). In particular, we showed that the PSi/C-dots hybrid can be used for designing label-free optical sensors using two orthogonal signals i.e., the reflectivity of the PSi nanostructure and the fluorescence of the confined C-dots. Moreover, we demonstrated that these two signal modalities can be simultaneously detected and collected. Importantly, we have also demonstrated the sensing performance of the PSi/C-dots system is superior when compared to that of the individual components.

Here, we present a new composite system comprising C-dots embedded within PSi-based Bragg reflector, designed to explore the interrelation between the optical properties of the confined C-dots and the PSi host. Bragg reflectors are constructed by a simple anodization process in which the current density is alternated between two distinct values in a stepwise manner and the resulting multilayered porous film displays alternating layers of high and low refractive index (Vincent, 1994; Pavesi and Dubos, 1997; Bisi et al., 2000). The optical thickness of the reflector layers is defined as λ/4, where λ is the center wavelength of the high reflectivity region (called photonic bandgap or stop band) over a desired spectral region (Bisi et al., 2000; Jane et al., 2009; Kilian et al., 2009; Pacholski, 2013). The position of the photonic bandgap is easily tuned by changing the electrochemical etching conditions (Sailor, 2011; Ning et al., 2014). The use of Bragg reflectors as the host matrix allows to control the propagation and distribution of the light emitted from the imbedded C-dots.

Bragg reflector/C-dots hybrids were constructed through a simple synthesis, in which a carbon precursor (such as glucose or sucrose solutions) was allowed to infiltrate into the PSi-based Bragg reflector matrix, and subsequent heating step generated the entrapped C-dots. We studied diverse types of C-dots with different emission spectra (blue, green, yellow and red) within various nanostructured Bragg reflectors with different photonic bandgap centers (425, 530, and 600 nm) and oxidation levels (freshly-etched, partially- and fully-oxidized). We demonstrate that by tuning the Bragg reflector photonic bandgap center to match the confined C-dots fluorescence emission, we can control the emission spectra properties. To the best of our knowledge, there is no study regarding optical properties of C-dots confined within photonic crystals. The present study can open up opportunities to design advanced nanomaterials with highly-tunable optical properties.

## Materials and methods

### Materials

Single-side polished and heavily-doped p-type Si wafers (0.001 Ω-cm resistivity, ‹100› oriented, B-doped) were obtained from Sil'tronix Silicon Technologies, France. Aqueous 48% hydrofluoric acid (HF) and ethanol (99.9%) were purchased from Merck, Germany. D-(+)-Glucose, Sucrose, Sodium tripolyphosphate, and O,O′-Bis (2-aminopropyl) polypropylene glycol-block-polyethylene glycol-block-polypropylene glycol1 (PEG-diNH_2_) were supplied by Sigma-Aldrich Chemicals, Israel.

### Fabrication of PSi bragg reflectors and fabry–pérot films

A two-step anodization process was applied to produce a highly-porous nanostructures with pores of 25–40 nm to allow accommodation the C-dots. First, a sacrificial layer was etched in the Si wafer at a constant current density of 385 mA cm^−2^ for 30 s (electrolyte composition: aqueous solution of HF and ethanol at 3:1 (v/v) ratio), using a ring of platinum as a counter electrode. Next, the resulting PSi layer was dissolved in an aqueous NaOH solution (0.1 M) followed by exposure to a solution composed of 1:3:1 (v/v/v) HF, ethanol and deionized water, respectively. Then, a second etching step was performed; the detailed etching conditions are summarized in Table 1. Finally, the freshly-etched films were thermally oxidized in a tube furnace (Thermo Scientific, Lindberg/Blue M™ 1,200°C Split-Hinge, USA) at 400°C or at 800°C for 1 h in ambient air, to form porous SiO_2_ (PSiO_2_) nanostructures.

**Table 1:** Etching conditions of the different PSi films and their structural characteristics (i.e., average pore diameter and porous layer thickness as determined by high-resolution Scanning electron microscopy, HRSEM).

**Photonic bandgap (nm)**	**Etching conditions (mA/cm**^**2**^**, s)**			**Porous film characteristics**			
				**Pore diameter**^a^**(nm)**		**Pore diameter^b^ (nm)**	**Thickness (nm)**
	**High refractive index layer**	**Low refractive index layer**	**No. of repeats**	**High refractive index layer**	**Low refractive index layer**	
425	77, 1.37	385, 0.48	40	25 ± 3	38 ± 7	50 ± 6	6,360 ± 20
530	77, 1.59	385, 0.56	40			
600	77, 1.93	385, 0.65	40			
Single layer	385, 30			42 ± 10		54 ± 10	4,920 ± 40

*The PSi Bragg reflectors with bandgap centers at 425, 530, and 600 nm were prepared from highly doped p-type single-crystal Si wafers anodized at alternating current densities of 77 and 385 mA cm^−2^. The etching time of each layer slightly differ from wafer to wafer, due to small variations in the wafer resistivity and the following oxidation process. PSi Fabry–Pérot films, termed as single-layers, were fabricated at a constant current density of 385 mA cm^−2^ for 30 s*.

a *Average pore diameter was measured from cross-sectional SEM images*.

b *Average pore diameter was measured from top-view SEM images*.

### Scanning electron microscopy

The morphology, i.e., thickness and average pore diameter, of the PSi Bragg reflectors and Fabry–Pérot thin films were characterized by high resolution scanning electron microscopy (HRSEM), using a Carl Zeiss Ultra Plus instrument, operated at an accelerating voltage of 1 keV.

### Fourier transform infrared (FTIR) spectroscopy

Freshly-etched and thermally-oxidized Bragg reflector films were characterized by FTIR spectroscopy at attenuated total reflectance (ATR) mode, utilizing a Thermo 6700 FTIR instrument, equipped with a Smart iTR diamond ATR device.

### *In situ* synthesis of c-dots within PSi films

Various C-dots (blue, green, yellow and red) were incorporated into the porous films by *in situ* synthesis within the nanostructures. Different carbonaceous precursor solutions were introduced onto the upper surface of the PSi film and allowed to infiltrate into the pores. Subsequently, the samples were placed in an oven and mildly heated. Table 2 summarizes the composition of the different precursor solutions and the pyrolysis conditions for each type of C-dots.

**Table 2:** Precursor aqueous solution composition and pyrolysis conditions for each type of C-dots.

**C-dots**	**Precursor solution concentration (mg mL^−1^)**	**Pyrolysis conditions**	
		**Temperature (^°^C)**	**Time (h)**
Blue	Glucose, 200	125	6
Green	Glucose, 400	150	6
Yellow	Sucrose, 400	150	6
Red	Glucose, 40 Na_5_P_3_O_10_, 0.4 PEG-diNH_2_, 20	150	7

### Synthesis of c-dots dispersions

Green C-dots dispersions, termed as “free” C-dots, were synthesized by a mild slow heating (at 150°C for 6 h) using the aqueous precursor (400 mg glucose in 1 mL of double-distilled-H_2_O) in a Teflon-lined autoclave chamber.

### Measurement of fluorescence emission spectra

The PSi/C-dot hybrid's fluorescence emission spectra were recorded at different excitation wavelengths using a spectrofluorimeter (FL920, Edinburgh Instruments, UK). The emission spectra were measured at different excitation wavelengths ranging from 300 to 600 nm.

### Confocal laser scanning microscopy (CLSM)

Following the *in situ* synthesis of C-dots within the PSi films, the resulting hybrids were characterized using an LSM 700 (Carl Zeiss, Germany) confocal laser scanning microscope (CLSM) connected to a Zeiss inverted microscope equipped with a Zeiss × 63 oil immersion objective. Laser lined at 405 and 555 nm were used to excite the PSi and the C-dots, respectively. Three-dimensional projection images of the hybrids were obtained using ZEN 2009 (Carl Zeiss, Germany) software; where z-scans in 0.5 μm increments were taken over a depth of ~8 μm and projected and stacked. Imaris software (Bitplane AG, Zurich, Switzerland) was used for subsequent image analysis.

### Transmission electron microscopy

The nanostructure of “confined” (extracted from the hybrids) and “free” (prepared in solution) C-dots was studied by a JEOL JEM-2100F high-resolution transmission electron microscope (HRTEM), at an accelerating voltage of 200 keV. Confined C-dots were collected from PSiO_2_/C-dots hybrids after dissolution of the PSiO_2_ matrix in a solution of HF and ethanol (3:1 v/v, respectively), followed by extraction of C-dots in absolute ethanol. Subsequently, the resulting C-dots solution was drop-casted on a graphene-coated copper grid.

### X-ray photoelectron spectroscopy (XPS)

Thermo Scientific ECSALAB X-ray photoelectron spectrometer with an AlKα x-ray source and a monochromator was used to characterize the “confined” (extracted from the hybrids) and “free” (prepared in solution) C-dots. The X-ray beam size was 500 μm and survey spectra were recorded with pass energy of 150 eV; high-energy resolution spectra were recorded with pass energy of 20 eV. XPS data was processed using the AVANTGE software.

### Measurement of interferometric reflectance spectra

Interferometric reflectivity spectra of the different PSi/C-dots hybrids were collected using a USB4000 (Ocean Optics, USA) charge-coupled device (CCD) spectrometer, which is fitted with a microscope objective lens coupled to a bifurcated fiber optic cable. A tungsten light source was focused onto the center of the sample surface with a spot size of 1–2 mm^2^; where both illumination of the surface and collection of the reflected light were performed along an axis coincident with the surface normal. The reflectivity data were recorded in a wavelength range of 350–850 nm, with a spectral acquisition time of 25 ms.

## Results and discussion

### Preparation and characterization of porous silicon bragg reflectors

**Figure 1:**
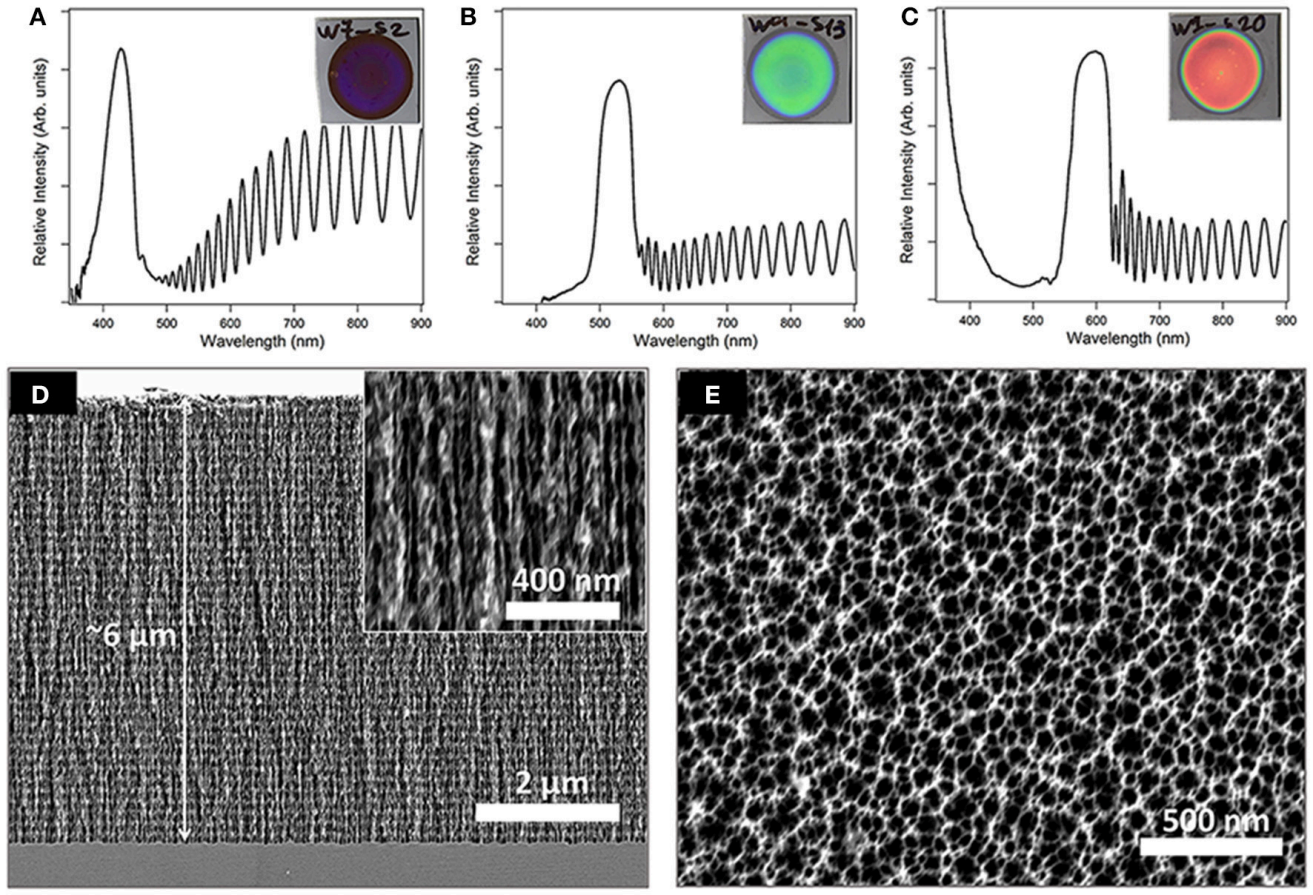
Upper panel presents images and corresponding reflectivity spectra of the different PSi Bragg reflectors with bandgap centers at **(A)** 425, **(B)** 530, and **(C)** 600 nm. Lower panel depicts characteristic HRSEM images of a PSiO_2_ Bragg reflector **(D)** cross-sectional and **(E)** top-view.

Porous Silicon (PSi) Bragg reflector films with photonic bandgap centers at 425, 530, and 600 nm were fabricated by Si anodization and the detailed etching conditions are summarized in Table 1 (Materials and Methods section). In general, 40 pairs of alternating high (n_H_ = 1.87) and low (n_L_ = 1.12) refractive index layers were constructed. Figures 1A–C presents characteristic images of the resulting PSi Bragg reflectors and their corresponding reflectivity spectra. The detailed structure of the films, in terms of their porous layer thickness and pore diameter, was studied by HRSEM. Figure 1D depicts cross-sectional micrographs of a typical PSiO_2_ Bragg reflector, revealing the periodic nanostructure, which consists of a series of thin layers of alternating high and low refractive indices. The thickness of the resulting porous layer is ~6.3 μm with interconnecting cylindrical pores (Bisi et al., 2000; Zhang, 2004), ranging in diameter from 25 to 40 nm. The average diameter of the pores' entrance is ~50 nm (see Figure 1E, top-view micrograph), which is somewhat larger than the bulk pores owing to the two-step anodization process. Table 1 summarizes the detailed structural features for the three types of Bragg reflector films.

In the next step, the freshly-etched PSi films were thermally oxidized at 400°C or at 800°C for 1 h (denoted as partially- and fully-oxidized PSi, respectively). The photonic bandgap centers of the Bragg reflectors were tuned to values of 425, 530, and 600 nm after the thermal oxidation step. In order to do so, the freshly-etched PSi Bragg reflectors were fabricated to higher photonic bandgap centers, while after oxidation the bandgap wavelengths were blue-shifted to the desired mentioned values. The chemical properties of the different porous films were characterized by FTIR-ATR spectroscopy and the obtained spectra are presented in Figure S1 (Supplementary Material). The spectrum of a freshly-etched PSi surface (Figure S1, trace A) depicts typical Si-H_x_ bending modes at 625, 661, and 920 cm^−1^ (Socrates, 2001; Xia et al., 2006). Two small peaks related to the Si-OH stretching modes are also observed at 820 and 883 cm^−1^ (Socrates, 2001; Xia et al., 2006). The latter are possibly attributed to the very thin native oxide layer that forms on the surface upon exposure to air. The spectrum of a partially-oxidized PSi surface (Figure S1, trace B) depicts small peaks that are related to Si-H_x_ bending modes at 625 and 900 cm^−1^. An intense broad peak at 1,056 cm^−1^, which is not observed for the freshly-etched surface, is ascribed to the Si–O–Si stretching mode (Socrates, 2001; Xia et al., 2006; Massad-Ivanir et al., 2011). Furthermore, an additional peak related to –(O_y_SiH_x_) vibration mode is observed at 802 cm^−1^ (Shtenberg et al., 2014). The spectrum of the fully-oxidized PSi surface (Figure S1, trace C) depicts a larger Si-O-Si stretching mode peak at 1,056 cm^−1^ and –(O_y_SiH_x_) vibration mode peak at 800 cm^−1^. The latter are in correlation with the disappearance of the Si–H_x_ peaks (at 625 and 900 cm^−1^), owing to complete oxidation of hydrogen-terminated Si species (Shtenberg et al., 2014).

### Synthesis of bragg reflectors/c-dots hybrid

**Figure 2:**
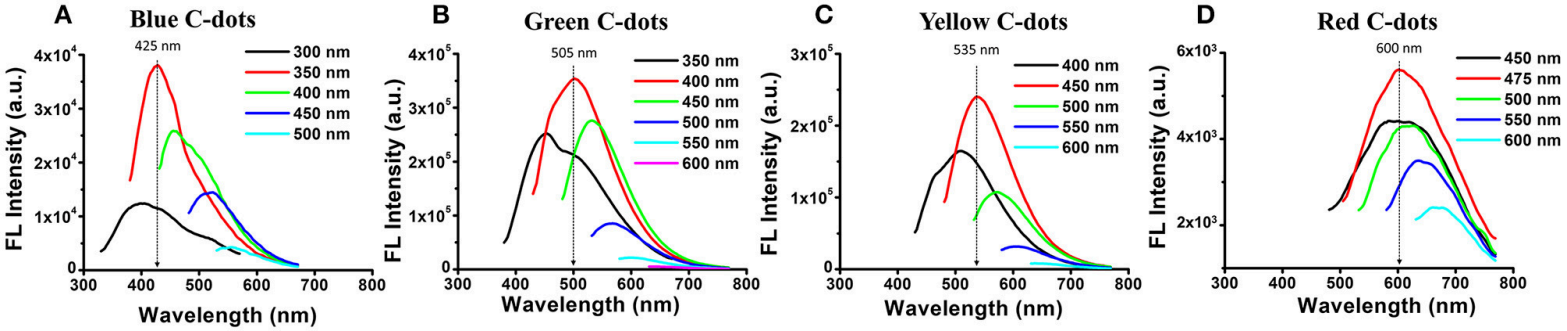
Fluorescence emission spectra at different wavelengths of **(A)** blue **(B)** green **(C)** yellow and **(D)** red C-dots confined within PSiO_2_ single-layer films (the color of the different spectra corresponds to different excitation wavelengths). The black arrows mark the maximum emission of the specific PSiO_2_/C-dots hybrid. Using these values, the photonic bandgap centers of the Bragg reflector hosts were further tuned to overlap.

PSi/C-dot hybrids were synthesized by allowing the carbonaceous precursor solution (see Table 2 for details) to infiltrate into the porous nanostructure, followed by a mild pyrolysis process. In order to tune the photonic bandgap centers of Bragg reflectors to overlap with the fluorescence emission of the C-dots, we first characterized the emission spectra of C-dots embedded within Fabry–Pérot PSiO_2_ thin films and representative results are presented in Figure 2 (for blue, green, yellow and red C-dots). According to obtained spectra, the etching conditions of the different Bragg reflectors were further tuned to adjust their photonic bandgap center to overlap with the wavelength at which maximum fluorescence emission from the C-dots was attained. These values are marked by arrows in Figure 2 and correspond to 425, 505, 535, and 600 nm of blue, green, yellow and red C-dots, respectively. In the next step, the C-dots precursors (see details in Table 2) were introduced into their corresponding PSi Bragg reflectors substrates. The latter included freshly-etched, partially-oxidized and fully-oxidized PSi and thus, three types of PSi/C-dots were prepared for each Bragg reflector.

### Characterization of the confined c-dots

The detailed nanostructure and composition of the C-dots, confined within the PSiO_2_ Bragg reflectors, were characterized by HRTEM and XPS, respectively. The confined C-dots were extracted from the PSiO_2_ by dissolving the porous scaffold in HF and compared to “free” C-dots, which were prepared in solution from the same carbonaceous precursors.

**Figure 3:**
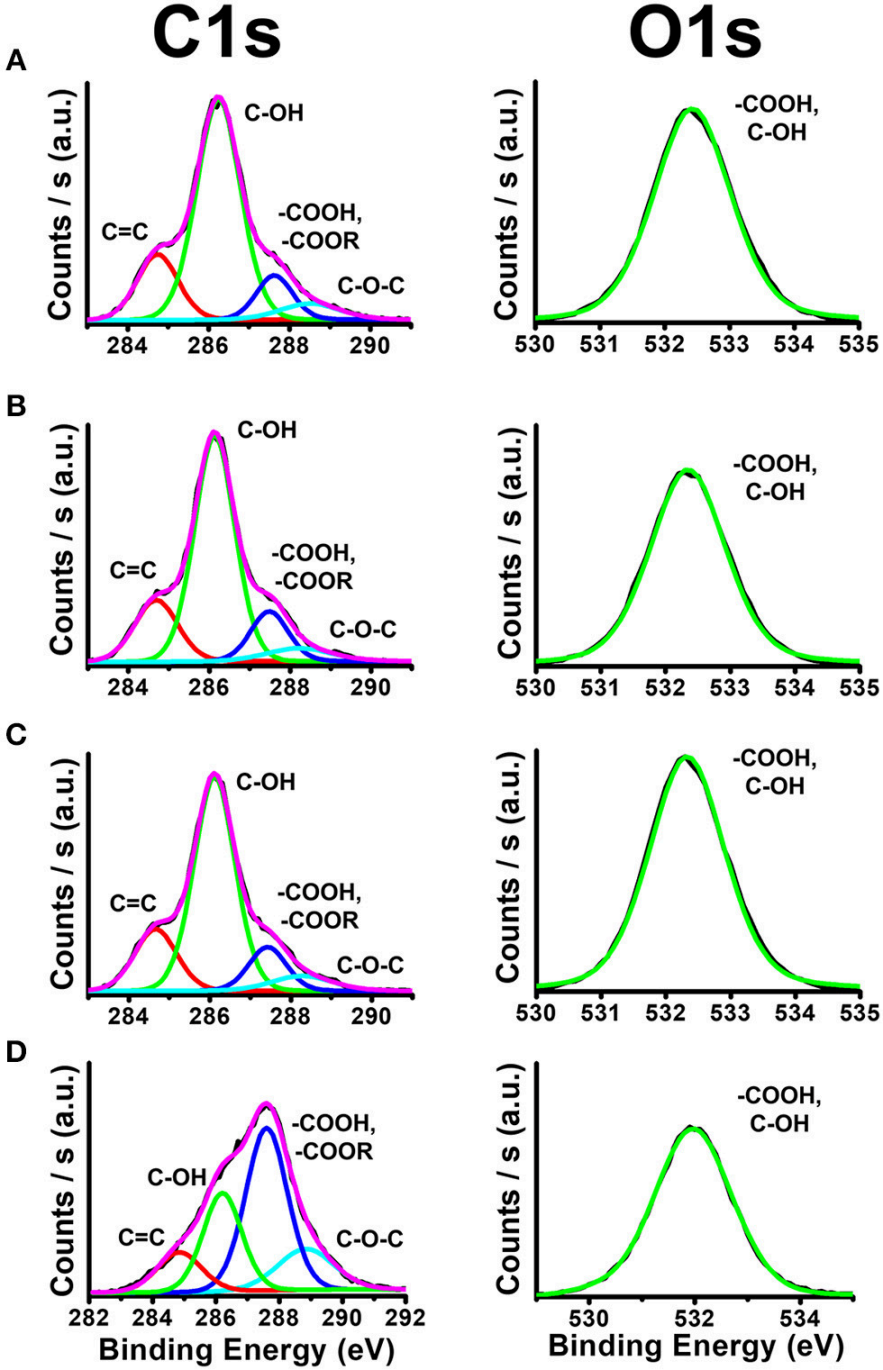
X-ray photoelectron spectra (XPS) showing the different functional groups on the C-dots surface. **(A)** C-dots extracted from PSiO_2_ Fabry–Pérot thin films, **(B,C)** C-dots extracted from PSiO_2_ Bragg reflectors with bandgap centers at 425 and 530 nm, respectively and **(D)** “free” C-dots synthesized in solution.

The XPS data shown in Figure 3 depicts the atomic species present in confined and “free” green C-dots (as representative C-dots). These results confirm minor differences between C-dots prepared *in situ* from confined precursor within the PSiO_2_ Bragg reflector matrix and C-dots synthesized in solution. Specifically, the deconvoluted C 1s spectrum displays peaks at 284.7 eV, corresponding to sp^2^ carbon atoms (C = C), 286.1 eV, assigned to C–OH groups and 287.6 eV, for –COOH and/or –COOR groups (Bhunia et al., 2016a,b; Massad-Ivanir et al., 2018). The O 1s spectrum shows peaks at 532.3 eV for O = C = OH and/or C–OH groups. The same XPS C1s and O1s signature peak positions were observed for C-dots extracted from PSiO_2_ Fabry–Pérot thin films (Figure 3A), C-dots extracted from PSiO_2_ Bragg reflectors with different photonic bandgap centers (Figures 3B,C) and “free” C-dots (Figure 3D). It is important to note that the “free” C-dots demonstrate the same XPS C1s and O1s signature peak positions. However, different deconvoluted XPS peak intensities were obtained, confirming that distinctive C-dots were formed within the porous nanostructures, in agreement with our previous work (Massad-Ivanir et al., 2018).

**Figure 4:**
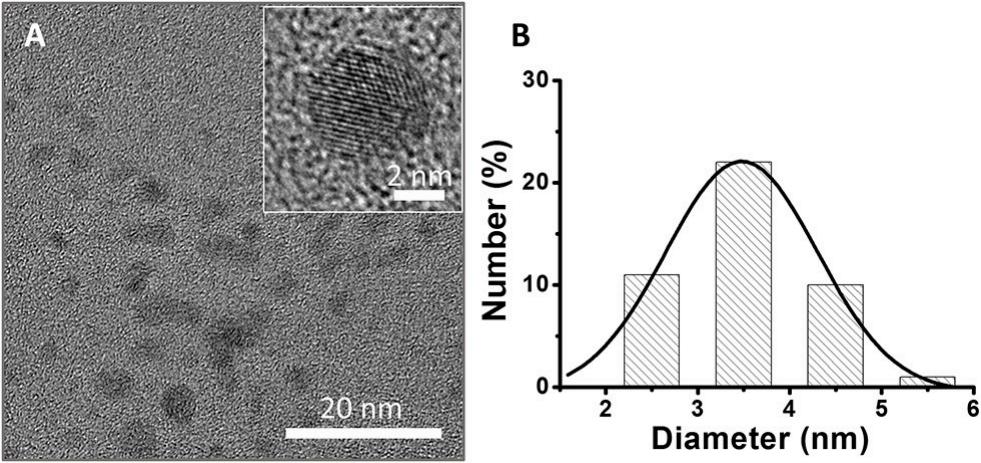
**(A)** TEM and HRTEM images of green C-dots extracted from the Bragg reflectors with bandgap center at 425 nm (scale bars: 20 and 2 nm, respectively). **(B)** Size distribution of extracted C-dots, inferred from the HRTEM experiment. Average size of 4 ± 2 nm.

The representative TEM and HRTEM images shown in Figures 4, S2 (Supplementary Material) reveal the structural features of green C-dots prepared *in situ* within the Bragg reflector nanoscale pores. The C-dots extracted from the different PSiO_2_ matrices (Bragg reflectors with bandgap centers at 425 nm, 530 nm and PSiO_2_ Fabry–Pérot thin film) exhibit a uniform spherical shape (see Figures 4, S2, S3, respectively) with a typical size of ~3.5 nm, as determined by size distribution analysis. The inset in Figure 4 (as well as insets in Figures S2, S3) depicts detailed nanostructure of the collected nanoparticles. The crystalline graphite cores of the extracted C-dots are clearly observed with an in-plane lattice spacing of 0.215 nm, corresponding to the [110] plane of graphite (Dolai et al., 2017). The “free” green C-dots, prepared in solution, exhibit an average diameter of ~3.2 nm and a similar nanostructure, see Figure S4 (Supplementary Material). These results confirm that the surface chemistry and size distribution of the confined C-dots are similar, regardless the nanostructures architecture and the location of Bragg reflector photonic bandgap center.

**Figure 5:**
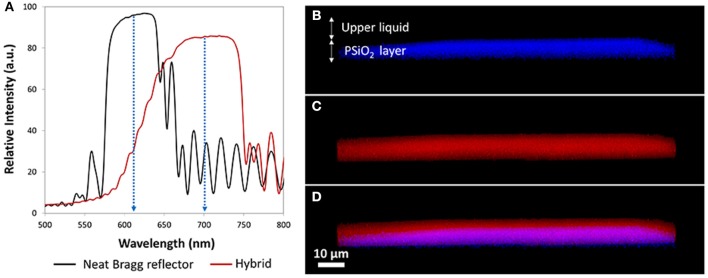
Reflectivity spectra of the PSiO_2_ (i.e., fully-oxidized) Bragg reflector before and after *in-situ* synthesis of the C-dots and the corresponding CLSM 3D projection images of the resulting PSiO_2_ Bragg reflector/C-dots hybrid. **(A)** Reflectivity spectra of PSiO_2_ Bragg reflector before (neat; black trace) and after (hybrid; red trace) *in situ* synthesis of red C-dots. **(B)** Photoluminescence of the PSiO_2_ Bragg reflector; **(C)** fluorescence signal of confined red C-dots; **(D)** combined view of **(A,B)**, demonstrates the location of the C-dots within the porous layer in the z-direction.

To validate the location of the C-dots within the porous layer, the reflectance spectra of the Bragg reflectors were recorded, before and after the C-dots synthesis. For partially- and full-oxidized PSi reflectors, significant red-shifts of the photonic bandgap, in the range of 75–125 nm, were observed following the *in situ* synthesis of the C-dots (Figure 5A), suggesting formation of the C-dots within the porous nanostructure. While, for the freshly-etched PSi reflectors, no change in the photonic bandgap was detected (Figure S5), indicating that the C-dots did not penetrate into the nanostructure. Confocal laser scanning microscopy (CLSM) is used as a complementary tool to follow the distribution of the C-dots within the oxidized layers and clearly reveal the dispersion of C-dots within the pores, see Figures 5B–D. Specifically, the blue photoluminescence (PL) signal (Figure 5B), which is ascribed to the PSiO_2_ scaffold (λ_ex_ = 405 nm, λ_em_ ≥ 420 nm, long pass filter; Sa'ar, 2009), allows for the analysis of the host matrix and can be spatially correlated to the fluorescence of the red C-dots (λ_ex_ = 555 nm, λ_em_ ≥ 560 nm, long pass filter) within the porous film (Figure 5C). Note that the C-dots fluorescence was recorded in z direction from the upper surface into the pores over a depth of ~8 μm, where z-scans in 0.5 μm increments were taken, projected and stacked as presented in Figures 5B–D. The overlay image (Figure 5D) confirms that fluorescence of the C-dots was observed throughout the entire depth of the porous scaffold over a distance of ~6 μm. These results also indicate that the laser beam penetrated into the porous layer and induced excitation of the embedded C-dots, resulting in a substantial emission from entire depth of the PSiO_2_. Residual fluorescence signals of the C-dots were also detected in a region slightly above the PSiO_2_ interface (Figure 5D), likely because of attachment of the nanoparticles to the PSiO_2_ surface.

### Optical properties of bragg reflector/c-dots hybrids

**Figure 6:**
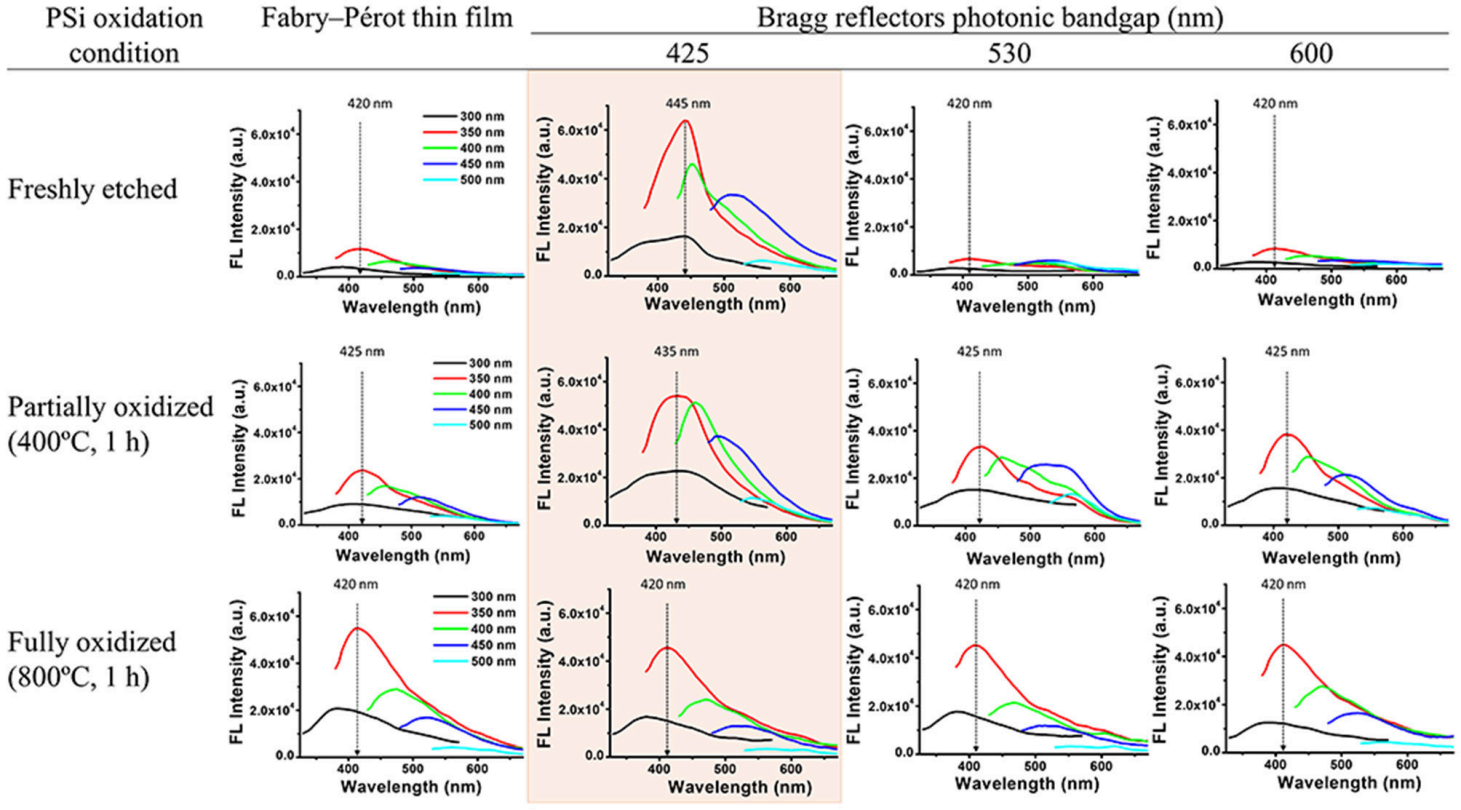
Fluorescence emission spectra of PSi/blue C-dots hybrids at different excitation wavelengths (indicated by the different colors). A comparison between different nanostructures (Fabry–Pérot thin film vs. Bragg reflectors with diverse bandgap centers) with varied oxidation states (none, partially and fully).

The optical properties of the confined C-dots were investigated while embedded within different PSi optical nanostructures (Fabry–Pérot thin film vs. Bragg reflectors with diverse photonic bandgap centers) at varied oxidation levels (none, partially, and fully). It is worth noting that the Bragg reflector hosts provide very high surface areas that are accessible to UV irradiation, thus capable of exciting the C-dot fluorescence. Figure 6 underscores the changes in emission spectra recorded for different PSi/blue C-dots hybrids. Importantly, the fluorescence emission spectra clearly depended upon the nanostructure architecture, the location of the Bragg reflector bandgap centers, and the oxidation level of the porous surface. Upon elevating the oxidation level, a decrease in the fluorescence quenching was apparent (i.e., the freshly-etched PSi induced the most significant quenching effect, which diminishes with oxidation). This phenomenon is ascribed to energy transfer between the Silicon and the C-dots, which results in the quenching of C-dots fluorescence signal (DeLouise Lisa and Ouyang, 2009). Moreover, enhancement, narrowing, and red shift (of 10–25 nm) of the emission spectra were achieved when the emission wavelength of the C-dots (~420 nm for blue C-dots) matched the photonic bandgap center of the nanostructure (Bragg reflector with bandgap center at 425 nm), see highlighted spectra in Figure 6. The enhancement of the C-dots emission spectra can be related to the high reflection band of the Bragg reflector, which reflect upwards the C-dots' fluorescence. This enhancement cannot occur when the Bragg reflector bandgap is far beyond the C-dots fluorescence emission. Similar behavior was demonstrated for QDs embedded within PSi-based Bragg reflectors (or deposited on top of PSi Bragg reflectors; Liu et al., 2015; He et al., 2017; Li et al., 2017). Narrowing and shift of the emission spectra were also observed in similar hybrid systems in which QDs were confined within the pores of PSi photonic crystals (Dovzhenko et al., 2018a,b). For fully oxidized surfaces, this effect is not observed and this behavior may be attributed to the poor contrast between the alternating layers. Moreover, in some cases, following the C-dots synthesis within the fully-oxidized Bragg reflectors, the characteristic reflectivity of the Bragg reflectors is distorted as presented in Figure S6 (Supplementary Material).

**Figure 7:**
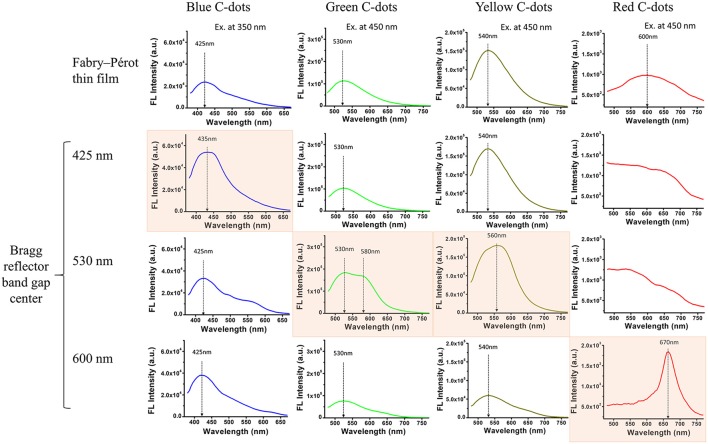
Fluorescence emission spectra of partially-oxidized PSi/C-dots hybrids upon excitation at different wavelengths. A comparison between different nanostructures (single layer vs. Bragg reflectors with diverse bandgap centers) with varied C-dots colors.

Next, all four types of C-dots were synthesized within the partially-oxidized PSi structures (reflectors and Fabry–Pérot thin films) and Figure 7 depicts the fluorescence emission spectra of these hybrids. It can be clearly seen that the fluorescence emission spectra depend upon the location of the respective Bragg reflector bandgap center. For blue C-dots, the fluorescence signal intensity was enhanced only when the emission wavelength of the C-dots (~420 nm) overlapped with the Bragg reflector bandgap center (at 425 nm), and a red shift of 10 nm was apparent, see highlighted spectrum in Figure 7 (left panel). This behavior was also observed for the yellow and green C-dots, as seen in the highlighted spectra in Figure 7 (middle panels). Notably, in the case of the PSi/green C-dots hybrids, the spectrum also broadened and a shoulder at 580 nm is apparent. The red C-dots exhibit the most pronounced enhancement and narrowing effect of all studied hybrids, as highlighted in Figure 7 (right panel). In addition, in the latter case, the C-dots fluorescence peak has red-shifted by ~70 nm. To summarize, the fluorescence intensity enhancement can be explained as spatial redistribution of the emitted light into a narrow cone normal to the surface (Baba et al., 1991; Qiao et al., 2010). Thus, the reflector's structure prevents the isotropic propagation of the C-dots fluorescence, and as a result their emission is preferentially directed toward the detector. Similarly, in previous studies on QDs embedded within PSi-based Bragg reflectors, a significant enhancement of the fluorescent emission was observed when their fluorescence peak overlaps with the reflector's bandgap (Liu et al., 2015; He et al., 2017; Li et al., 2017). Essentially, the Bragg reflector modified the optical mode density of the C-dots confined within the porous nanostructure, therefore enhancing emitted wavelengths inside the resonance wavelengths (Baba et al., 1991; Yamamoto et al., 1991; Qiao et al., 2010).

**Figure 8:**
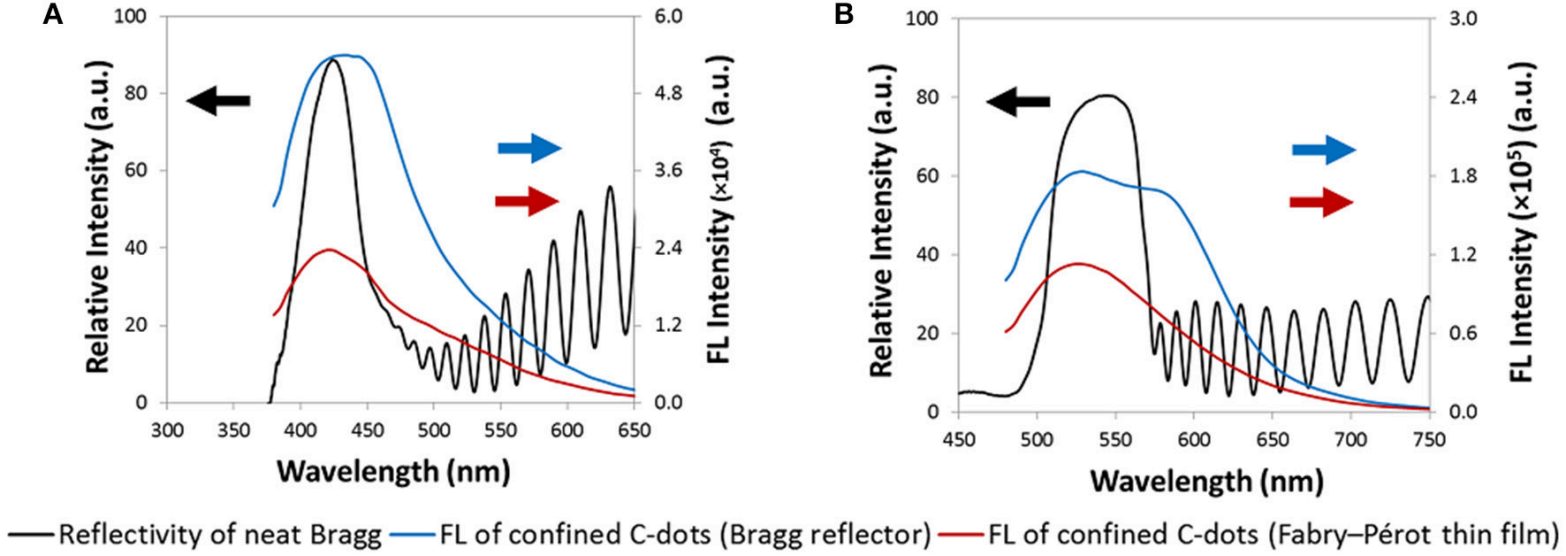
Reflectivity spectra of Bragg reflectors (black trace) and the corresponding fluorescence emission spectra of the confined C-dots (blue trace). **(A)** Photonic bandgap at 425 nm; blue C-dots. **(B)** Photonic bandgap at 530 nm; green C-dots. For comparison, the fluorescence emission spectra of the C-dots embedded within Fabry–Pérot thin films are also presented (red trace).

To further study the specific effect of the PSi Bragg reflectors reflectivity on the fluorescence properties of the confined C-dots, we present in Figure 8 both the reflectivity spectra of neat Bragg reflectors (with no C-dots) and the corresponding fluorescence emission spectra of the confined C-dots. For comparison, the fluorescence emission spectra of the C-dots embedded within Fabry–Pérot thin films are also presented. For blue C-dots, the significant enhancement of the fluorescence intensity (in comparison to that observed for hybrids based on Fabry–Pérot thin films) corresponds to the right edge of the photonic bandgap (Figure 8A). Also, for green C-dots the fluorescence enhancement is manifested by a new shoulder at 580 nm, which overlaps with right edge of the host's photonic bandgap (Figure 8B). Figure S7 (Supplementary Material) presents the respective spectra also for yellow and red C-dots, exhibiting a similar trend. Dovzhenko et al. (2015) have also shown that the PL spectrum of QDs embedded within PSi is affected by the shape of the Bragg reflectivity spectrum. Specifically, the QDs PL was enhanced at a wavelength which corresponds to the edge of the photonic bandgap of the Bragg reflector host. This behavior is apparent also in the studied hybrids.

In conclusion, a hybrid system consisting of a PSi Bragg reflector matrix and encapsulated fluorescent C-dots enabled unique tunability of the C-dots' fluorescence, depending upon coupling between the C-dots' optical properties and the Bragg reflectors' bandgaps. Thus, by careful design of the porous host, in terms of the Bragg reflector photonic bandgap and the PSi oxidation state, the PL properties of the embedded C-dots can be modulated and fine-tuned. Notably, we have found that the fluorescence emission spectrum of the confined C-dots is dependent upon the porous host nanostructure architecture, the shape and the location of the Bragg reflector photonic bandgap, and the oxidation level of the porous surface. For the best of our knowledge, this is the first time that FL optical properties of C-dots are characterized within PSi Bragg reflector matrix. The ability to this hybrid system to specifically modulate the photophysical properties of C-dots may advance the design of sophisticated nanomaterials for sensing and bioimaging.

## Author contributions

NM-I fabricated and characterized the Bragg reflectors, conducted the HRSEM analyses and carried out the reflectivity measurements. SB synthesized and characterized the Bragg reflectors/C-dots hybrids, conducted the HRTEM and XPS analyses. All authors discussed the results and implications at all stages. ES and RJ have conceived the research, designed the study, and analyzed data. All authors wrote the manuscript.

### Conflict of interest statement

The authors declare that the research was conducted in the absence of any commercial or financial relationships that could be construed as a potential conflict of interest.
