# Supplementary material for: Porous Silicon Bragg Reflector/Carbon Dot Hybrids: Synthesis, Nanostructure, and Optical Properties
Source: Front Chem. 2018 Nov 23;6:574. doi: 10.3389/fchem.2018.00574 (PMC6265313; doi:10.3389/fchem.2018.00574)
Supplement: Supplementary file 1 [file Data_Sheet_1.docx]

Supplementary Material

Porous Silicon Bragg reflectors/carbon dot hybrids: Synthesis, nanostructure and optical properties

**Naama Massad-Ivanir^1§^, Susanta Kumar Bhunia^2,3§^, Raz Jelinek^3,4*^, Ester Segal^1,5*^**

*** Correspondence:** Prof. Ester sagel: [esegal@technion.ac.il](mailto:esegal@technion.ac.il) and Prof. Raz Jelinek: [razj@bgu.ac.il](mailto:razj@bgu.ac.il)

**
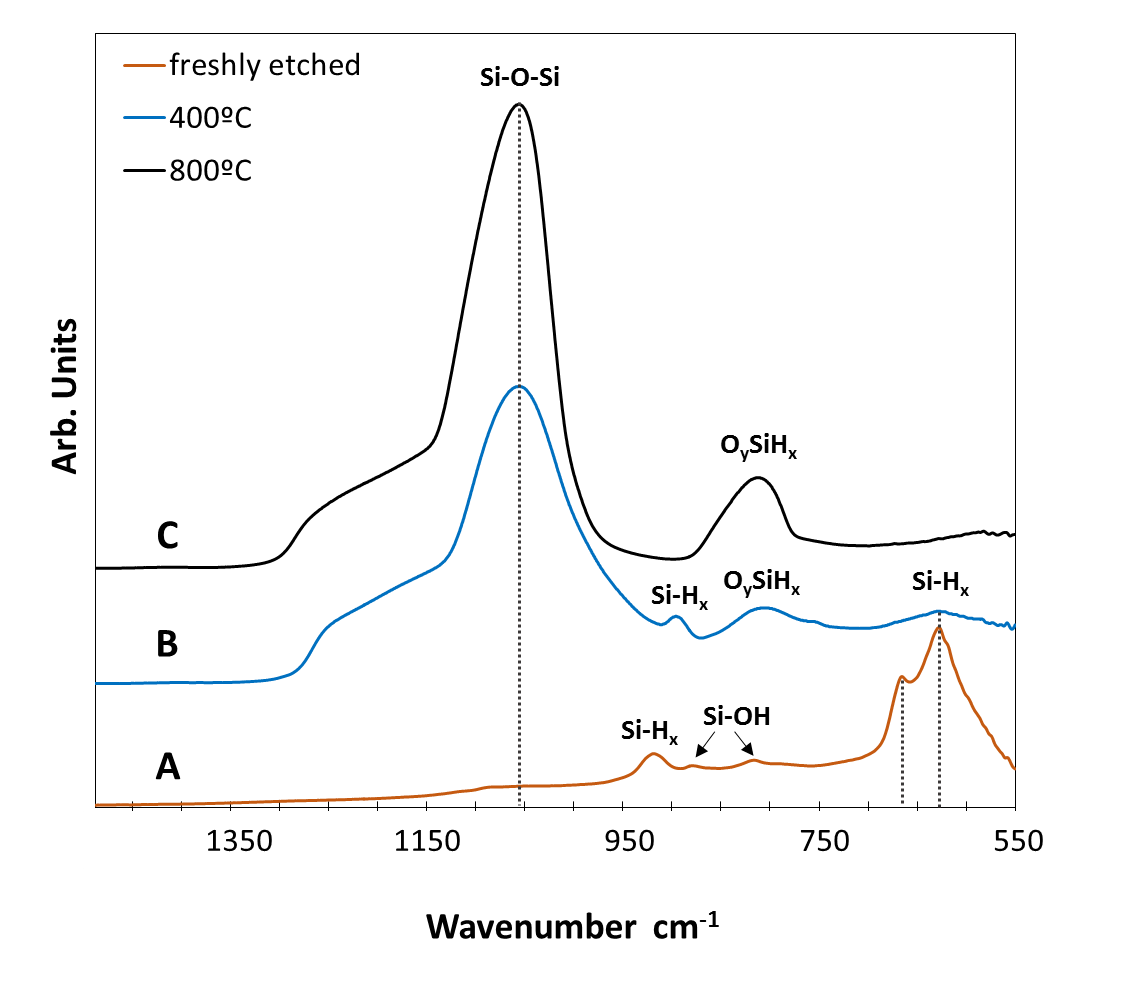
**

**Supplementary Figure 1.** FTIR-ATR spectra of the different surfaces: (A) freshly-etched PSi, (B) partially-oxidized PSi (400ºC, 1 h), and (C) fully-oxidized PSi (800ºC, 1 h).


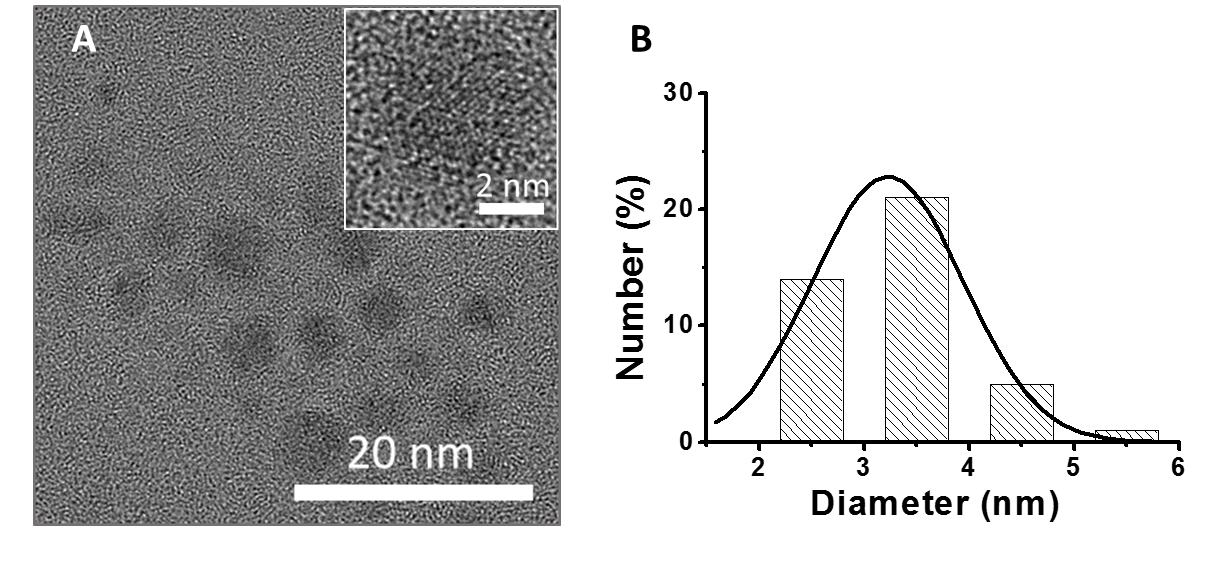


**Supplementary Figure 2.** (A) TEM and HRTEM images of green C-dots extracted from the Bragg reflectors with photonic bandgap center at 530 nm. (B) Size distribution of the extracted C-dots, inferred from the HRTEM experiment. Average size of 3.3 ± 1.7 nm.


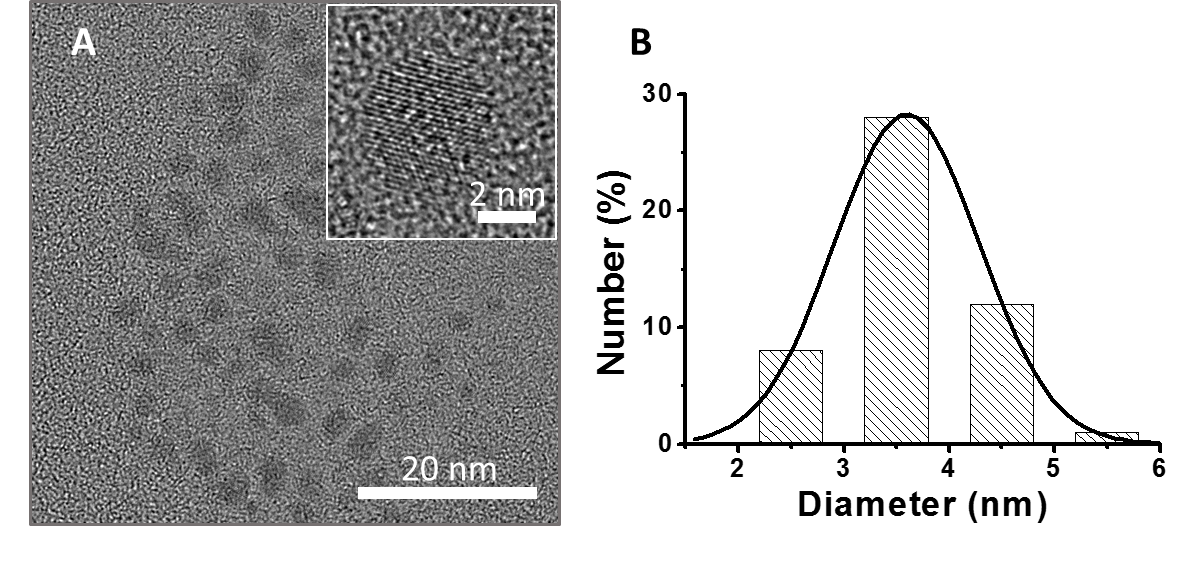


**Supplementary Figure 3.** (A) TEM and HRTEM images of green C-dots extracted from a PSiO_2_ thin film. (B) Size distribution of extracted C-dots, inferred from the HRTEM experiment. Average size of 3.6 ± 1.5 nm.


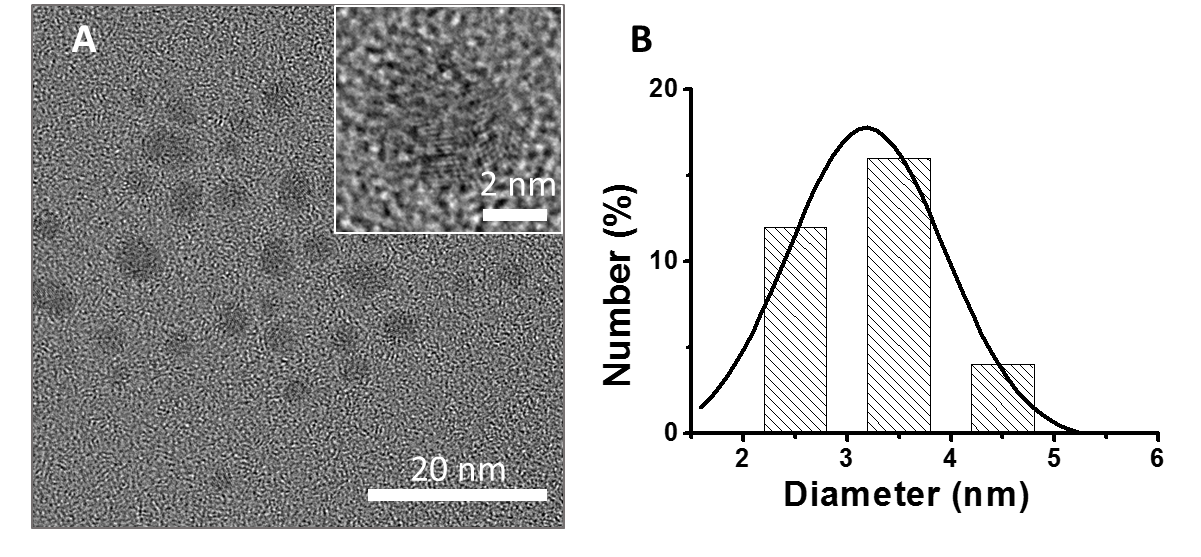


**Supplementary Figure 4.** (A) TEM and HRTEM images of ‘free’ green C-dots prepared in solution. (B) Size distribution of ‘free’ C-dots, inferred from the HRTEM experiment. Average size of 3.2 ± 1.2 nm.

**
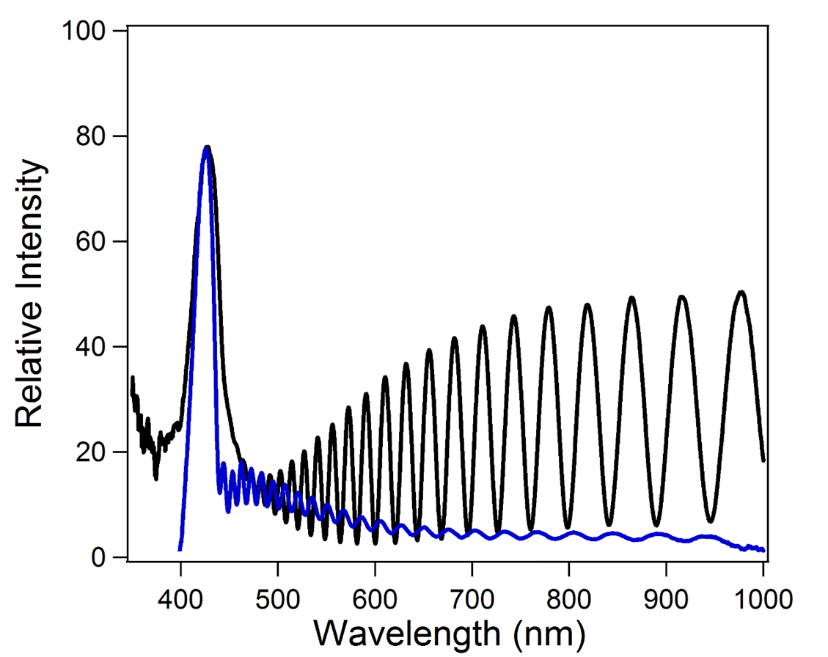
**

**Supplementary Figure 5.** Reflectivity spectra of freshly-etched PSi Bragg reflector before (black trace) and after (blue trace) in-situ synthesis of C-dots within the pores.

**
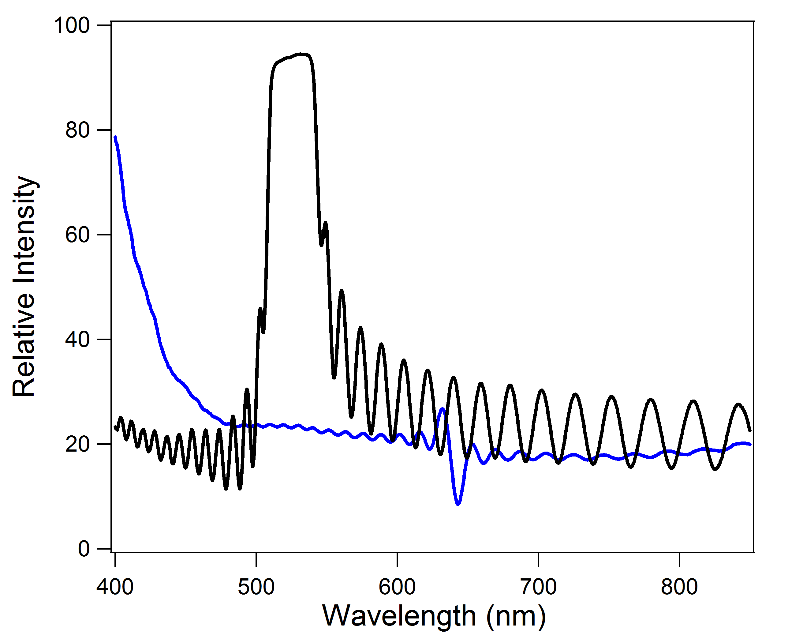
**

**Supplementary Figure 6.** Reflectivity spectra of fully-oxidized PSi Bragg reflector before (black trace) and after (blue trace) in-situ synthesis of C-dots within the pores.

**
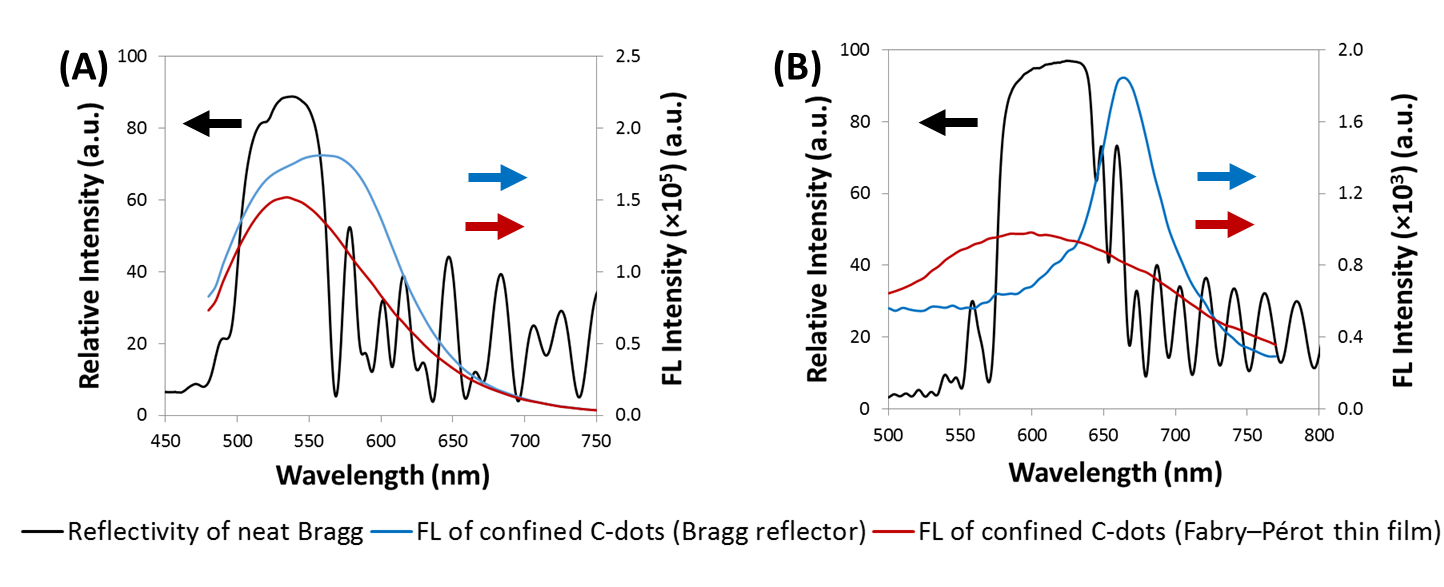
**

**Supplementary Figure 7.** Reflectivity spectra of Bragg reflectors (black trace) and the corresponding fluorescence emission spectra of the confined C-dots (blue trace). (A) Photonic bandgap at 530 nm; yellow C-dots. (B) Photonic bandgap at 600 nm; red C-dots. For comparison, the fluorescence emission spectra of the C-dots embedded within Fabry–Pérot thin films are also presented (red trace).
